# Quantitative proteomics suggests changes in the carbohydrate metabolism of maize in response to larvae of the belowground herbivore *Holotrichia parallela*

**DOI:** 10.7717/peerj.9819

**Published:** 2020-08-27

**Authors:** Yu Pan, Shiwen Zhao, Zhun Wang, Xiao Wang, Xinxin Zhang, Yunshuo Lee, Jinghui Xi

**Affiliations:** 1 College of Plant Science, Jilin University, ChangChun, China; 2 Changchun Customs Technology Center, ChangChun, China

**Keywords:** *Holotrichia parallela*, TMT, Maize roots, Glycolysis

## Abstract

The larvae of *Holotrichia parallela*, a destructive belowground herbivore, may cause yield losses of up to 20% in maize in a typical year. To understand the protein-level mechanisms governing the response of maize to this herbivore, tandem mass tag (TMT) quantitative proteomics was used for the comparative analysis of protein abundance in the maize roots after *H. parallela* larval attack. A total of 351 upregulated proteins and 303 downregulated proteins were identified. Pathway enrichment analysis revealed that the differentially abundant proteins (DAPs) were most strongly associated with carbohydrate and energy metabolism pathways, such as glycolysis, pentose phosphate pathway and fructose and mannose metabolism. Most glycolysis-related proteins were significantly induced. In addition, *H. parallela* larval attack decreased the glucose concentrations in the roots. This study demonstrates that maize can manipulate carbohydrate metabolism by modifying glycolysis and pentose phosphate pathway response to root-feeding herbivorous attackers. The results of this study may help to establish a foundation for further functional studies of key protein-mediated responses to *H. parallela* larvae in maize.

## Introduction

Plants have evolved many complex and ingenious defense mechanisms, such as direct defense and indirect defense, in response to insect herbivore attack. Direct defense responses involve the production of secondary metabolites and insecticidal proteins, which can reduce herbivore development and survival (Erb & Reymond, 2019). Indirect defense responses primarily involve the release of volatile organic compounds that can attract natural enemies of herbivores (such as predators and parasitoids) (Turlings & Erb, 2018).

Well-documented examples of plant antiherbivore compounds include alkaloids, nicotine, glucosinolates and benzoxazinoids with a wide range of insecticidal and antifeedant activities (Wu & Baldwin, 2010; Steppuhn et al., 2004; Zhou et al., 2015; Robert et al., 2017). However, herbivores have also evolved resistance mechanisms to adapt to toxic compounds (Lindigkeit et al., 1997). As a consequence, plants employ alternative tolerance strategies by the induction of photosynthesis and reallocation of resources, enabling them to increase tolerance to various herbivores (Machado et al., 2013; Carmona & Fornoni, 2013). For example, leaf herbivory *Ectropis Oblique* can significantly reduce non-structural carbohydrates, such as glucose, fructose and sucrose, in damaged tea leaves (Yang et al., 2019). Accumulating evidence has demonstrated that plants respond to herbivores by decreasing the concentrations of carbohydrate resources in damaged tissues, a process termed ‘herbivory-induced resource sequestration’ (Schwachtje et al., 2006; Holland, Cheng & Crossley, 1996). The GAL83-silenced tobacco that the b-subunit of the sucrose non-fermenting-related kinase (SnRK1), tolerates *Manduca sexta* attacked by increasing carbon in the roots (Schwachtje et al., 2006). However, attack by the leaf herbivore *Manduca sexta* decreased sugar and starch concentrations in the roots of *Nicotiana attenuata* (Machado et al., 2013). Clearly, the connection between carbohydrate metabolism and tolerance merits further investigation if we are to understand the role of primary metabolism in plant defensive strategies.

Maize (*Zea mays L.*) is one of the most important food and industrial crops worldwide. The world’s maize production reached 1.1 billion tons in 2018, and China, the country with the second largest maize yield worldwide, produced 0.26 billion tons (http://faostat3.fao.org/browse/Q/QC/E). During the lifetime of the plant, different parts are subjected to attacks from various groups of insects (Meihls, Kaur & Jander, 2012). *H. parallela* larvae are considered one of the most economically important pests attacking maize in China (Yi et al., 2018). *H. parallela* larvae can cause considerable damage, with yield losses ranging from 10 to 20% in a typical year (Ju et al., 2017). Because *H. parallela* larvae live in the soil, it is difficult to control them using traditional pesticides. Achieving a better understanding of the genetic and molecular mechanisms underlying the defense of maize against *H. parallela* larvae is important for developing resistant maize varieties and devising other strategies for controlling pests.

The changes in the transcriptome of maize in response to *H. parallela* larvae feeding has been investigated and has demonstrated potential as a means of elucidating the mechanisms governing induced defense (Pan et al., 2020). However, in maize attacked by *H. parallela* larvae, the changes in biological processes at the protein level have not been determined. The protein levels are more realistic compared to the mRNA levels because they are functional and post-translational processes result in the production of different protein isoforms (Hegde, White & Debouck, 2003). An iTRAQ-based method and a 2-DE approach have been used to explore the response of maize leaves to infestation with the herbivore *Mythimna separata* and the Asian corn borer, respectively (Qi et al., 2016; Zhang et al., 2015). However, several studies have focused on proteomic analysis of maize responses to belowground herbivores. Therefore, we used TMT-based proteomics to compare quantitative changes in the protein levels in maize roots to elucidate the possible defense mechanism induced by *H. parallela* larval attack.

## Material and Methods

### Plant material and insect treatment

Maize plants were sown in plastic pots (height, 20 cm; diameter, 15 cm) containing a mixture of pasteurized field soil and sand (3:1), as previously described (Pan et al., 2020). The original maize seedlings were supplied by Professor Yaping Yuan from Jilin University. The soil was excavated from the field of Changchun in Jilin Province (latitude 50°54′19″N, longitude 125°15′45″E) and was sieved through a 2-mm mesh before use. The seedlings were grown in a climate chamber (22 ± 2 °C, 70% relative humidity, 16 h:8 h light:dark). Thirteen days later, plants with two expanded primary leaves were used for the experiments (Erb et al., 2009). *H. parallela* larvae were collected from a local maize field in Changchun, China, and were reared with maize seedling roots at room temperature until use. For the proteomics analysis of maize seedlings, plants were either infested with one third-instar *H. parallela* larvae or left herbivore-free. After 24 h of infestation, the herbivores were gently removed, and the plants were harvested. The timing of this step was chosen based on previous studies showing a strong induction of defenses after 24 h of infestation (Marti et al., 2013; Glauser et al., 2011). The roots and leaves from the harvested plants were carefully excised from the shoots and weighed immediately to determine their fresh weight. Then, the roots were wrapped in aluminum foil, immediately frozen in liquid nitrogen, and stored at −80 °C until use.

### Protein extraction

Protein extraction was performed using a modified method (Isaacson et al., 2006). Briefly, the root samples from maize seedlings were frozen in liquid nitrogen and pulverized. The samples were mixed with five volumes of TCA/acetone (1:9) by vortexing and incubated at −20 °C for 4 h. The supernatant was discarded after centrifugation at 6000 g for 40 min. The precipitant was washed three times with pre-cooling acetone and was air-dried. The pellet was dissolved in SDT buffer containing 4% SDS, 150 mM Tris-HCl, 1mM DTT, (pH 8). The lysate was sonicated then boiled for 15 min. Finally, the supernatant was filtered through 0.22-µm filters after centrifugation at 14,000 g for 40 min. The supernatant was quantified with the BCA Protein Assay Kit (Bio-Rad, USA). The quality of the protein sample was measured by SDS-PAGE.

### TMT labeling and HPLC fractionation

Two hundred micrograms of protein for each sample was incorporated in 30 µl SDT buffer. The detergent, DTT and other low-molecular-weight components were removed using UA buffer (8 M urea, 150 mM Tris-HCl at pH 8.0) by repeated ultrafiltration. Then, 100 µl iodoacetamide (100 mM IAA in UA buffer) was added to block reduced cysteine residues, and samples were incubated for 30 min in darkness. The filters were washed with 100 µl UA buffer three times and then washed with 100 µl 100 mM triethylammonium bicarbonate (TEAB) twice. Finally, the protein from each sample was digested with trypsin (Promega, USA) at a protein/trypsin ratio of 40:1 overnight at 37 °C. The peptides were labeled using TMT six-plex isobaric label reagent (Product No: MAN0011639, Thermo Fisher Scientific, USA) according to the manufacturer’s instructions. The sample peptides were fractionated using alkaline reverse-phase HPLC with an Agilent 300 Extend C18 column. The wavelength used for the detection of peptides was 250 nm. Briefly, the peptides were first separated into 80 fractions over 80 min using a gradient of 2–60% acetonitrile in 10 mM ammonium bicarbonate (pH 10). Then, the peptides were combined into 18 fractions and dried by vacuum centrifugation.

### LC-MS/MS analysis

Each fraction was dissolved in 0.1% formic acid and loaded onto an Acclaim PepMap 100 reversed-phase C18 trap column (Thermo Fisher, Shanghai, China). Separation of peptides was performed using an EASY-nLC system and then subjected to tandem mass spectrometry Q EXACTIVE (Thermo Fisher Scientific) for data-dependent acquisition (DDA) detection by nano-electrospray ionization. The main parameters for MS analysis were collision energy, 30 eV; precursor scan range, 300–1,800 m/z; dynamic exclusion duration, 40.0 s; automatic gain control for full MS target, 3e6.

### Database search

The MS/MS raw data were searched using the Mascot search engine embedded into Proteome Discoverer 1.4. The main Mascot search parameters were as follows: carbamidomethyl (C), TMT6/10plex (N-term), TMT6/10plex (K) as fixed modifications, and oxidation (M) as variable modifications. Additionally, 20 ppm of the peptide mass tolerance and 0.1 Da of the fragment mass tolerance was allowed. To reduce the probability of false peptide identification, only peptides with significance scores at the 95% confidence interval with a Mascot probability identity threshold were considered to be identified. The global false discovery rate was <1%, and each confident protein identification involved at least one unique peptide. Proteins quantitatively detected in all six samples were used to further analyze the abundance change between the control and treatment. Differentially abundant proteins (DAPs) were identified based on the following criteria: *P*-values smaller than 0.05 and a mean relative abundance > 1.2 or <0.83. The mass spectrometry proteomics data have been deposited to the Proteome EXchange Consortium via the PRIDE partner repository with the dataset identifier PXD018939.

### Bioinformatics and annotations

To determine the functional characterization of proteins, proteins and differentially abundant proteins (DAPs) were mapped with Gene Ontology (GO) annotation (http://www.geneontology.org). All proteins were grouped into three major categories: biological processes, cellular components and molecular functions. The Kyoto Encyclopedia of Genes and Genomes (KEGG) (http://www.kegg.jp/kegg/pathway.html) was employed to predict each DAP’s main metabolic pathway classification. SubLoc (Guo et al., 2004) was applied to predict the subcellular localization of each identified protein.

### Herbivory-induced reconfiguration of primary metabolism in roots

To evaluate whether belowground herbivory reconfigures the primary metabolism in roots, we analyzed the contents of soluble sugar. Sucrose and hexose contents were determined using a sucrose, fructose and glucose assay kit (Mlbio, Shanghai, China) following the manufacturer’s instructions. The concentrations of glucose, fructose and sucrose were calculated using the calibration curves generated from corresponding standard solutions.

### Statistical analysis

The experiments were performed with three biological replicates, and plant materials from three seedlings were pooled for each biological replicate. The results are presented as the mean ± standard deviation (SD). One-way analysis of variance (ANOVA) with Tukey’s test was conducted on the data, and *p*-values < 0.05 were considered to be significant.

## Results

### Global proteomic changes in maize after *H. parallela* larvae infestation

**Figure 1:**
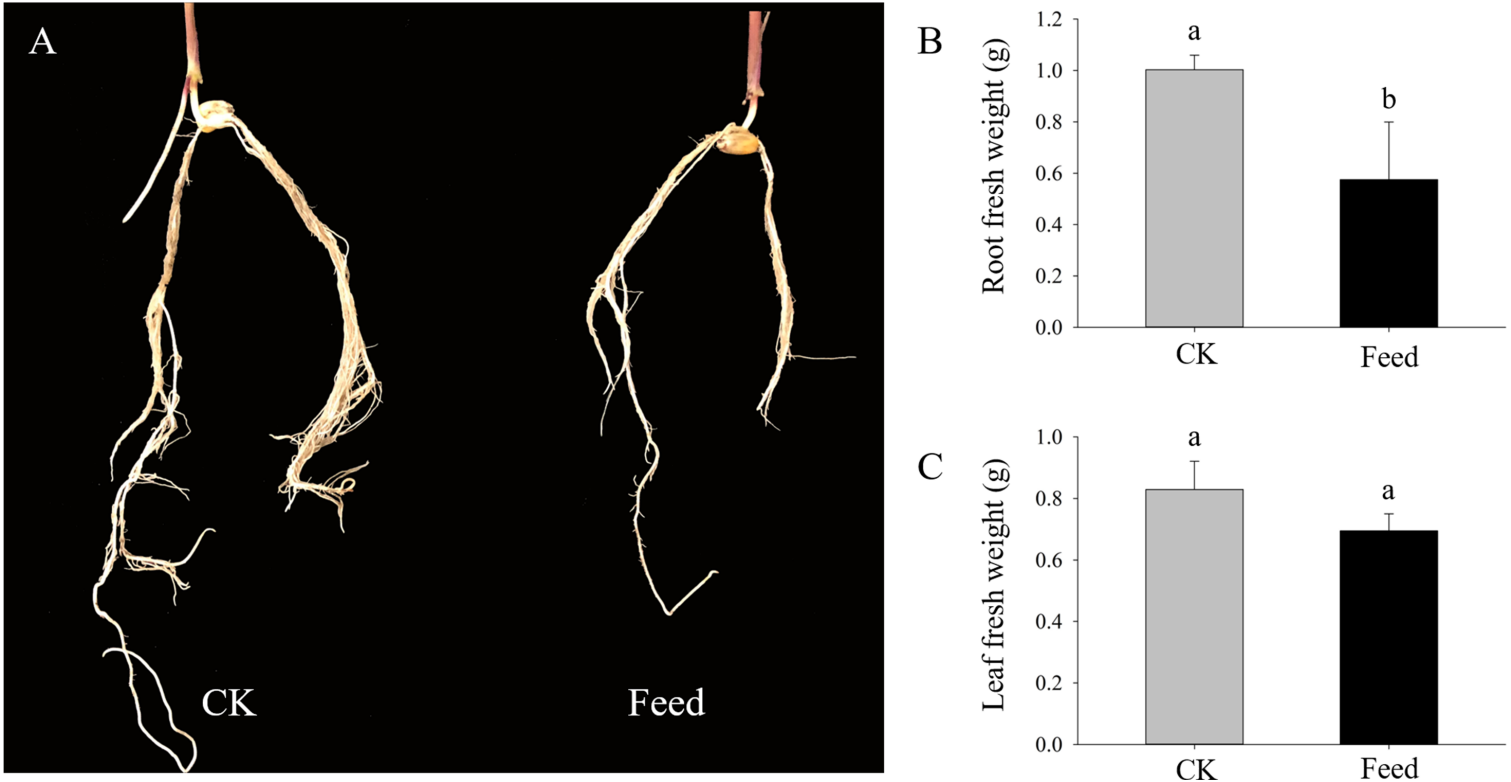
Growth parameters of maize seedlings under control and *H. parallela* larvae-stressed conditions. Data are presented as the mean ± standard deviation. CK: control; FEED: *H. parallela* larvae attacked. (A) Representative photograph of maize roots after *H. parallela* larvae attacked. (B) Root fresh weight (g). (C) Leaf fresh weight (g).

**Figure 2:**
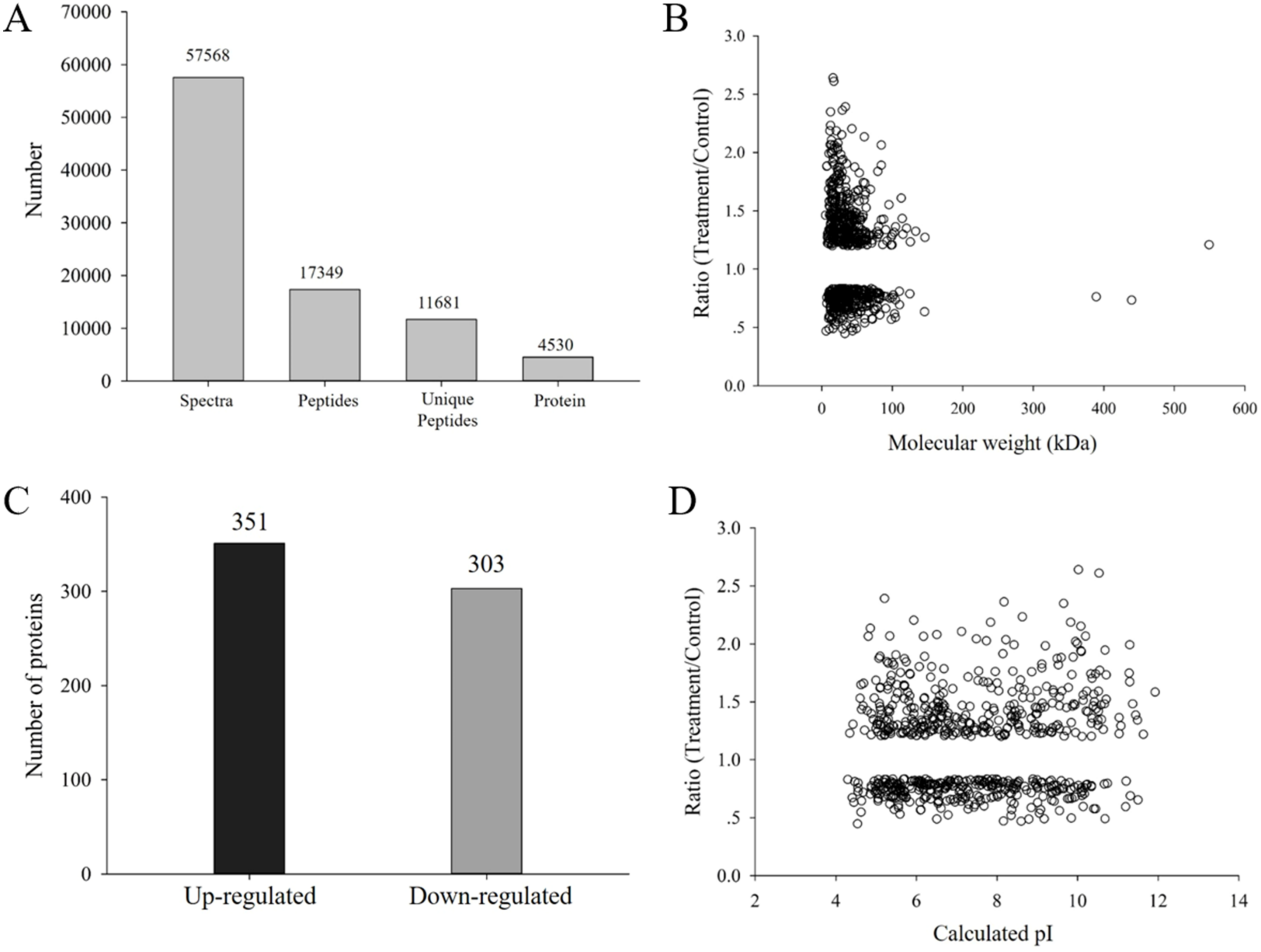
(A) Spectra, peptides, unique peptides and proteins identified from TMT proteomics; (B) distribution of fold changes and molecular masses for DAPs; (C) statistics of upregulated and downregulated DAPs in response to *H. parallela* larvae attack; (D) distribution of fold changes and isoelectric points for DAPs.

*H. parallela* larvae infestation significantly affected the fresh masses of maize roots and leaves, on average, 42.6% and 16.2% lighter compared to controls, respectively (Fig. 1). To investigate the changes in protein abundance in response to *H. parallela* larval attack in maize roots, comparative proteomics-based Tandem Mass Tag (TMT) labeling technology was conducted. After processing MS/MS spectra in Mascot software, a total of 57,568 spectra were generated from TMT analysis, yielding 17,349 matched peptides, 11,681 unique peptides, 4,530 matched proteins and 3,229 quantified proteins (Fig. 2A).

According to a criterion of 95% significance and a 1.2-fold cutoff, a total of 654 proteins were identified as DAPs between the attacked roots and the control roots. Among these DAPs, 351 and 303 were identified as upregulated and downregulated proteins, respectively, with fold changes of 0.44 to 2.63 and protein masses of 1.59 to 549.34 kDa (Fig. 2B). All detailed information is listed in Table S1.

### Enrichment analysis of DAPs under *H. parallela* larval attack

**Figure 3:**
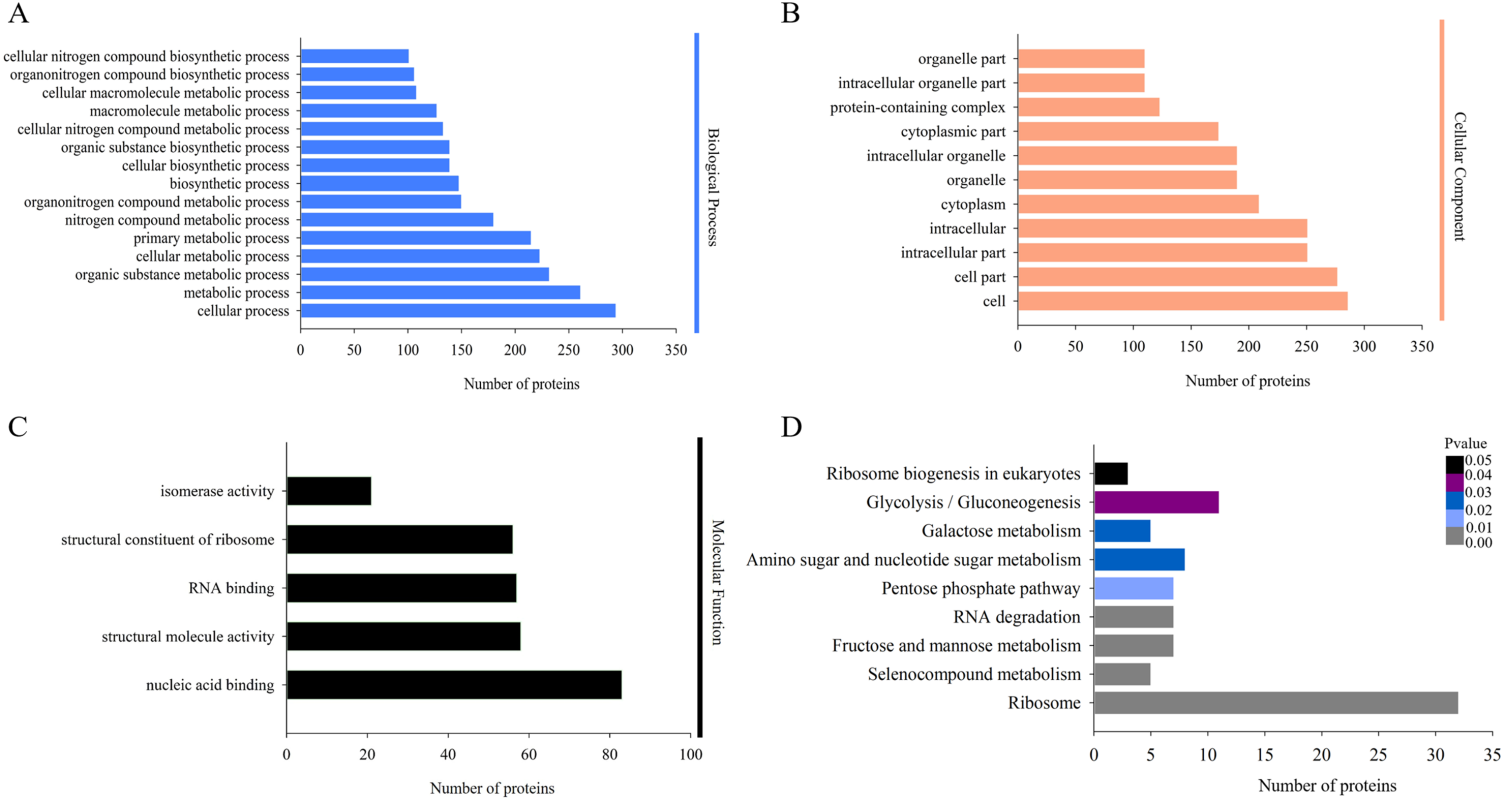
Gene Ontology (GO) and Kyoto Encyclopedia of Genes and Genomes (KEGG) analysis of DAPs. GO analysis of all identified DAPs. All proteins were classified by GO terms based on their biological process (A), cellular component (B) and molecular function (C). (D) KEGG analysis of DAPs. Different colors indicate the range of *p*-values for KEGG pathways.

The biological functions of the DAPs could also be identified by their GO annotations. DAPs were divided into three categories: cellular component, biological process and molecular function. For the ‘biological process’ category, the DAPs were enriched in several GO terms, such as ‘cellular process’, ‘metabolic process’, ‘organic substance metabolic process’, and ‘cellular metabolic process’ (Fig. 3A). In the ‘cellular component’ category, most of the enriched proteins were related to ‘cell’, ‘cell part’, and ‘intracellular’ terms (Fig. 3B). For the ‘molecular function’ category, the highly enriched proteins were associated with ‘nucleic acid binding’, ‘structural molecule activity’, and ‘RNA binding’ (Fig. 3C).

Proteins in the same pathway presumably perform their biological function collectively. Pathway enrichment analysis using the KEGG database was performed to characterize the biological function of *H. parallela* larvae-affected proteins. Among the 654 identified DAPs, 222 (33.94%) DAPs were assigned to 66 specific KEGG pathways. The majority of DAPs were associated with ribosome (32 DAPs), glycolysis/gluconeogenesis (11 DAPs), amino sugar and nucleotide sugar metabolism (8 DAPs), fructose and mannose metabolism (7 DAPs), pentose phosphate pathway (7 DAPs) and RNA degradation (7 DAPs) (Fig. 3D).

### DAPs involved in carbohydrate and energy metabolism

Carbohydrate and energy metabolism are one of the most basic metabolic pathways, and its mainly physiological function is to provide energy and a carbon source for many reactions and processes inside cells. Twenty-three DAPs involved in carbohydrate and energy metabolism were identified in maize roots responding to *H. parallela* larval attack, including proteins associated with glycolysis, gluconeogenesis, pentose phosphate pathway, and fructose and mannose metabolism (Table 1). The subcellular localization of most identified proteins and the other proteins related to carbohydrate and energy metabolism was cytoplasmic and mitochondrial, except for B6TT00 in the extracellular space (Table 1). Among these DAPs, 15 proteins were upregulated subsequent to *H. parallela* larvae in maize, with a ratio ranging from 1.214 to 1.679, including three dehydrogenases, two epimerases, two aldolases, two kinases and one protein from each of the isomerase, uridylyltransferase, diphosphorylase, mutase, galactosidase and uncharacterized protein families. Another 8 proteins, that is, two proteins in the dehydrogenase family, two phosphofructokinases, two mannosidases, one endochitinase and one uncharacterized protein, were downregulated subsequent to *H. parallela* larvae treatment, with a highest ratio of 0.611 being observed for mannan endo-14-beta-mannosidase 2.

**Table 1:** Identification of the DAPs involved in carbohydrate metabolism.

No.^a^	Description^b^	MW^c^ (kDa)	pI^d^	Local^e^	Fold change^f^	Pathway^g^
C0PHV6	Uncharacterized protein	63.00	5.71	Cytoplasmic	1.527	A, C, D, E
B6T6S5	Glucose-6-phosphate 1-epimerase	36.60	6.70	Cytoplasmic	1.464	A
B4G0K4	Phosphoglycerate kinase	42.41	5.87	Cytoplasmic	1.450	A
B4FS87	Glyceraldehyde-3-phosphate dehydrogenase	36.52	6.89	Cytoplasmic	1.284	A
B4FR32	Glyceraldehyde-3-phosphate deHaseN1	53.25	7.20	Cytoplasmic	1.242	A, C
B4FWP0	Fructose-bisphosphate aldolase	38.43	7.30	Cytoplasmic	1.235	A, B, C
B6TII5	Pyruvate kinase	55.28	8.06	Mitochondrial	1.214	A
B4G191	Uncharacterized protein	50.49	6.04	Cytoplasmic	0.798	A
B6U6D5	ATP-dependent 6-phosphofructokinase	58.94	7.87	Mitochondrial	0.761	A, B, C, E
B4FGJ4	Pyruvate dehydrogenase E1 component subunit alpha	42.72	8.19	Mitochondrial	0.743	A
C0HFI5	ATP-dependent 6-phosphofructokinase-2	57.26	7.85	Mitochondrial	0.671	A, B, C, E
B4FHJ2	Xylose isomerase	53.95	5.66	Cytoplasmic	1.239	B
B4FR89	Phosphomannomutase	28.11	6.23	Cytoplasmic	0.833	B, D
K7W6W9	GDP-mannose 4,6 dehydratase 2	41.28	8.10	Mitochondrial	0.702	B, D
B4F8R2	Mannan endo-14-beta-mannosidase 2	50.90	7.46	Mitochondrial	0.611	B
B4FRC9	Aldolase-type TIM barrel family protein	46.22	7.33	Cytoplasmic	1.460	C
B6TSB3	Glucose-6-phosphate 1-dehydrogenase	57.59	6.77	Cytoplasmic	1.335	C
B4FAD9	UTP–glucose-1-phosphate uridylyltransferase	52.15	5.36	Cytoplasmic	1.679	D, E
B4FBC2	GDP-mannose 35-epimerase	42.94	6.42	Cytoplasmic	1.466	D
K7UEK4	UDP-N-acetylglucosamine diphosphorylase 2	54.82	6.62	Cytoplasmic	1.419	D
P80607	Probable UDP-arabinopyranose mutase 1	41.18	6.13	Cytoplasmic	1.259	D
B6TT00	Endochitinase PR4	28.55	5.35	Extracellular	0.795	D
B4F9X0	Alpha-galactosidase	45.13	6.01	Cytoplasmic	1.296	E

**Notes.**

a No., Accession number.

b Description, Protein name obtained using Maize database from UniProtKB.

c MW, theoretical molecular weight.

d pI, theoretical isoelectric point.

e Local, localization category.

f Fold change is presented as the mean ± SE of three biological replicates.

g Pathway, Pathway name that protein involved in the KEGG. A: Glycolysis / Gluconeogenesis, B: Fructose and mannose metabolism, C: Pentose phosphate pathway, D: Amino sugar and nucleotide sugar metabolism, E: Galactose metabolism.

### Carbohydrate concentrations

**Figure 4:**
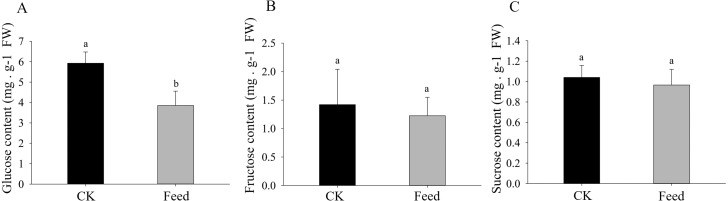
Glucose (A), fructose (B), and sucrose (C) contents in healthy or infested maize roots. Mean values ± SE are presented (mg g^−1^ of fresh weight). Different letters indicate significant differences (*p* < 0.05).

*H. parallela* larval attack led to a strongly reduced glucose concentrations in the roots compared to controls (Fig. 4). Although root fructose levels followed the same trend, these changes were not significant. The root sucrose contents in the attacked roots remained unaltered (Fig. 4).

## Discussion

Proteomics enabled us to screen out related proteins in response to *H. parallela* larvae attacking on a large scale to further elucidate the defense mechanisms in the maize root. However, few proteomic analyses of the defenses induced in maize in response to *H. parallela* larval attack have been reported. In this study, a TMT-based proteomic method was used to analyze the changes in protein abundance between control and *H. parallela* larvae-attacked seedlings.

**Figure 5:**
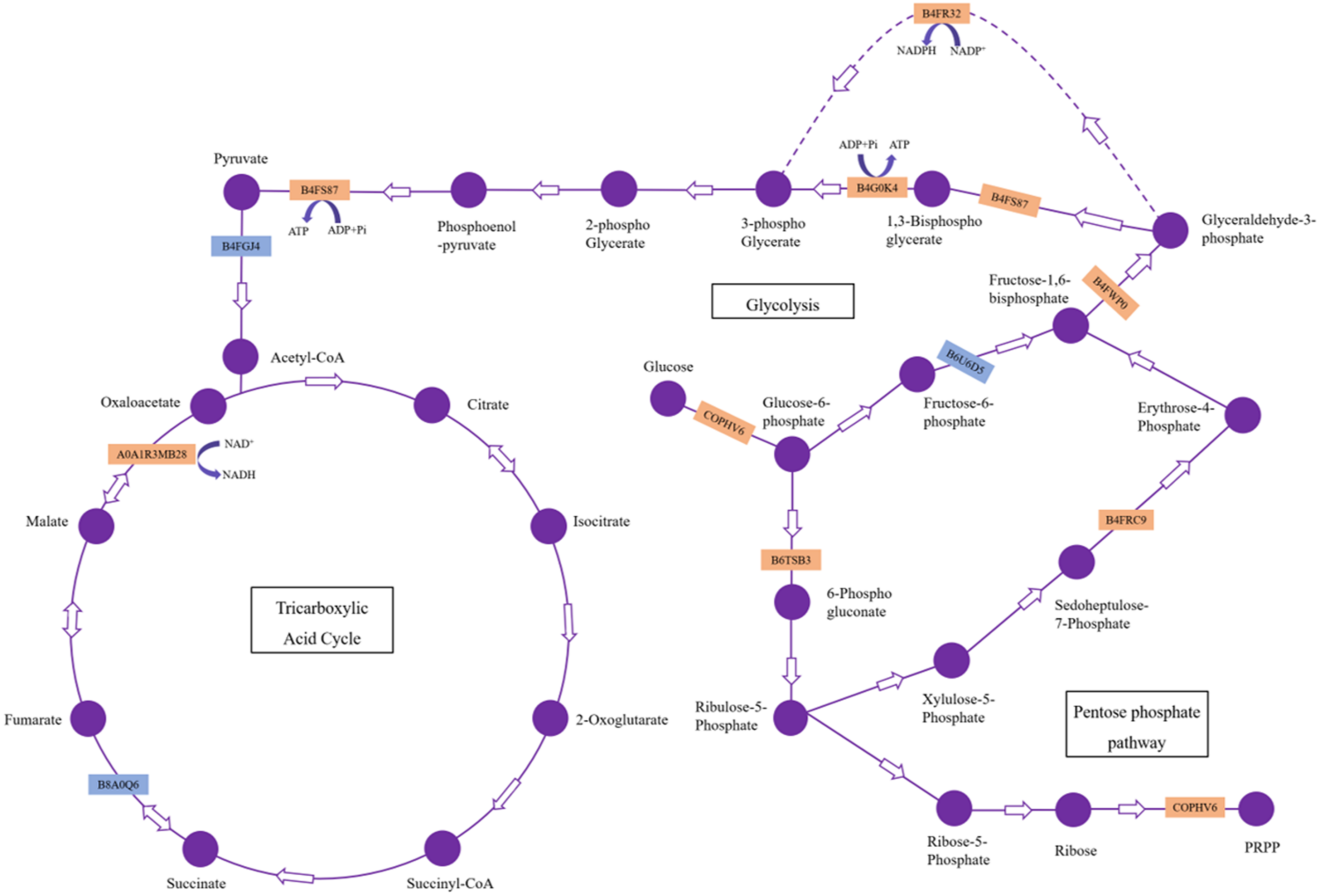
DAPs involved in carbohydrate metabolism pathways. The upregulated and downregulated DAPs identified in this study are indicated in the orange box and blue box, respectively.

Our results showed 23 DAPs involved in carbohydrate and energy metabolism, which is one of the most basic metabolic pathways, and its primary physiological role is to provide energy and a carbon source. Most of these DAPs were significantly upregulated, which indicated that *H. parallela* larval attack activated metabolic pathways, such as glycolysis, leading to abnormal carbohydrate metabolism and an insufficient supply of critical substrates (Fig. 5). These upregulated proteins included glyceraldehyde-3-phosphate dehydrogenase (GAPDH), phosphoglycerate kinase (PGK), and pyruvate kinase (PK), which affect the production of the energy source glucose in maize. Among these enzymes, GAPDH is reported to be an essential enzyme and converts glyceraldehyde-3P compounds to the corresponding glycerate-1,3-2P (Rius et al., 2006). The overexpression of the GAPDH gene in rice controlled the excessive accumulation of H_2_O_2_ and alleviated oxidative stress (Zhang et al., 2011). Wounding was observed to cause a rapid local and systemic ROS burst in *Arabidopsis* (Miller et al., 2009). Our results also confirmed that maize ROS-indicative proteins, such as superoxide dismutase (B4F925), catalase (K7UGM3), and peroxidase (C0P4K4), were activated in response to *H. parallela* larval attack (Table S1). We suggest that GAPDH alleviates ROS-induced cell damage in maize roots. In addition, GAPDH promotes the outward transfer of intermediate products, thereby providing more raw materials for subsequent synthetic reactions. PK is one of the primary rate-limiting enzymes of glycolysis pathways and catalyzes the final step of glycolysis, converting phosphoenolpyruvate to pyruvate (Turner, Knowles & Plaxton, 2005). This process is irreversible. Considerable evidence has indicated that herbivorous insect infestation influences carbohydrate and energy metabolism in plants. Carrillo, Rubiales & Castillejo (2014) reported the induction of the glycolysis pathway after pea aphid feeding on pea plants. The activation of genes and enzymes involved in glycolysis to produce more energy in rice-small brown planthopper interactions has also been shown (Dong et al., 2017). In addition, two key enzymes involved in the pentose phosphate pathway, glucose-6-phosphate 1-dehydrogenase and the aldolase-type TIM barrel family protein, were significantly upregulated after *H. parallela* larval attack. Carbohydrate anabolism was repressed, while carbohydrate catabolism was induced, in response to methyl jasmonate (MeJA) treatment in *Arabidopsis* (Chen et al., 2011). MeJA plays a critical role in plant defense against pathogens and insects by regulating the expression of defense-related genes (Browse, 2006). These results indicate that the upregulation of these proteins may have led to increased energy production through glycolysis and the pentose phosphate pathway to resist *H. parallela* larval attack.

Meanwhile, we also observed that the contents of glucose were strongly decreased in the maize root after infestation with *H. parallela* larvae. Infestation with the belowground herbivore *Delia radicum* caused a strong decrease in total sugars in the roots of broccoli and turnip, primarily due to decreases in glucose and sucrose (Pierre et al., 2012). Similarly, attacked maize roots accumulated less glucose than healthy plants following *D. virgifera* infestation (Christelle et al., 2012). These experiments indicated that plants respond to herbivore attack by decreasing the contents of carbohydrates in the roots. This phenomenon may be caused by plants facing increased energetic demands to support the production of inducible defenses in response to insect feeding. To cope with this challenge, many plants respond to herbivore attack by promoting the local catabolism of energy compounds (Zhou et al., 2015). For instance, a transcriptomic study investigating Arabidopsis and four different insect herbivores showed the increased expression of invertases and genes encoding enzymes involved in degrading carbohydrates (Appel et al., 2014). On the other hand, a lower carbohydrate content may hamper herbivore performance by reducing the nutritive value of plant tissue. For example, the weight gain of both the root herbivore cucumber beetle (*Diabrotica balteata*) and the rice water weevil (*Lissorhoptrus oryzophilus*) was significantly decreased in a jasmonate-deficient rice (*Oryza sativa*) mutant, despite the higher removal of root biomass. This effect was correlated with an herbivore-induced reduction in carbohydrate pools in rice roots (Lu et al., 2015). Therefore, the results of our study demonstrate that maize may manipulate carbohydrate metabolism against the larvae of the root herbivore *H. parallela* by activating glycolysis and pentose phosphate pathway. The capacity to reprogram carbohydrate metabolism after herbivore attack is important for plant survival and represents a complementary strategy for resistance to herbivores. However, the molecular basis of plant tolerance to herbivory via the regulation of carbohydrate metabolism remains poorly studied and merits further investigation.

## Conclusions

The DAPs expressed in response to attack by *H. parallela* larvae primarily belong to the glycolysis and pentose phosphate pathways, suggesting that maize activates the carbohydrate metabolism to respond to herbivore attack.

##  Supplemental Information

10.7717/peerj.9819/supp-1 Supplemental Information 1 Detailed information on the identified protein

10.7717/peerj.9819/supp-2 Supplemental Information 2 Raw data for identified protein, peptides

## Additional Information and Declarations

### Data Availability

The following information was supplied regarding data availability: The raw data is available in the Supplementary Files and at ProteomeXchange: PXD018939.
